# Supplementary material for: Cold spells over Greenland during the mid-Pliocene Warm Period
Source: Nat Commun. 2025 Feb 22;16:1877. doi: 10.1038/s41467-025-56996-3 (PMC11847007; doi:10.1038/s41467-025-56996-3)
Supplement: Supplementary file 1 — Supplementary Information [file 41467_2025_56996_MOESM1_ESM.docx]

**Supplementary material**

1. **Geology of Greenland terranes**

The geology of Greenland remains largely unexplored and poorly characterized due to the extensive coverage of the Greenland Ice Sheet (GIS), which conceals most of the land surface. This extensive ice cover, comprising approximately 80% of Greenland's area, limits direct geological investigations and the collection of rock samples, thereby compromising a comprehensive understanding of the region's subsurface geological framework and evolution. Greenland’s terranes that are possible source areas of sediments for our studied site on Eirik Drift are the Archean block (AB), Nagssugtoqidian Mobile Belt (NMB), Ketilidian Mobile Belt (KMB) and the Paleogene volcanism (PV). The AB (ε_Nd(0)_ mean=-32.4, median=-31; ^207^Pb/^206^Pb mean=0.94, median=0.95)^1^ encompasses rocks strongly deformed during the Archaean but almost unaffected by Proterozoic or later orogenic activities^2^. The NMB (ε_Nd(0)_ mean=-30.5, median=-32.3; ^207^Pb/^206^Pb mean=0.90, median=0.89)^1^ consists of reworked Archaean gneisses, early Proterozoic supracrustal rocks and intrusive rocks, which deformation and metamorphism peak occurred ca. 1850 Ma, when large parts of the belt underwent granulite facies metamorphism^2^. The KMB (ε_Nd(0)_ mean=-20.7, median=-22.1; ^207^Pb/^206^Pb mean=0.74, median=0.73)^1^ is composed of early Proterozoic sedimentary rocks and basalts^2^. The center of the KMB is dominated by the juvenile early Proterozoic granitic rocks of the Julianehåb batholith (1854-1795 Ma)^3^). Thus, although the tectonic evolution of both NMB and KMB is mostly constrained to the Palaeoproterozoic, NMB rocks have a higher crustal residence time, and differ in terms of ε_Nd(0)_ values and ^207^Pb/^206^Pb ratios from KMB. The areas dominated by the PV (ε_Nd(0)_ mean=4.5, median=5.8; ^207^Pb/^206^Pb mean=0.86, median=0.85) are composed of flood basalts and mafic plutons of ~55 Ma^4^, associated with sea-floor spreading.

1. **Sortable silts and biogenic carbonate fluxes**

During warm intervals, biogenic carbonates add to detrital supplies. Throughout most of the sequence, the calcite and grain size data are relatively well correlated, with maximums values during interglacial intervals marked by an increased Western Boundary Undercurrent (WBUC; as recorded by SS_mean_ bottom current strength proxy) and enhanced biogenic carbonate fluxes^5^. A few outlier values are linked to the maximum biogenic carbonate fluxes (Fig. S1) of the likely warmer ~3060 to ~3090 ka interval in the Blake-Mizen et al.'s age model (2019)^6^. Outliers reflect enhanced coccolithophorids production^5^ and do not change the interpretation regarding bottom current strength. SS_mean_ (µm) and mean grain size (µm) covary with SS(%) and sand (%) as shown in Fig. S2, below. Sand (%) measurements (>63µm fraction) from Blake-Mizen et al. (2019)^6^ were performed after carbonate removal and depict the same pattern of our samples (increased coarseness during interglacials), see Fig. S3.

Figure S1: Correlation of calcite (%) vs sortable silt mean size (µm). The outliers, in red, correspond to the peaking CaCO_3_-values of the 3060-3090 ka interval (see Fig. 3, main text).

.

 Figure S2: Correlation between SS_mean_ (µm) and SS(%), and mean grain size (µm) and sand (%).


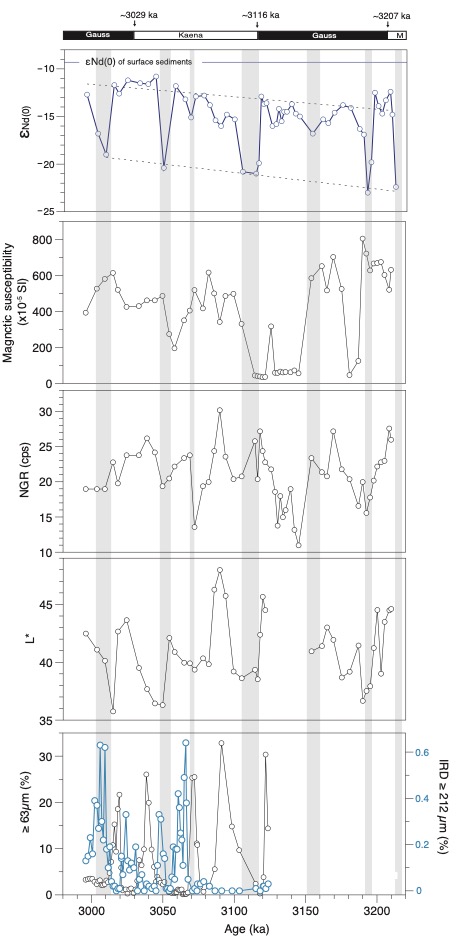


Figure S3: Additional measurements (shipboard) and ice rafted debris from site U1307. From top to bottom; Paleomagnetic record of core U1307^7^ (M = Mammoth) and approximate age of reversals^7,8^; Sediment ε_Nd(0)_ values (this work); Magnetic susceptibility; Natural gamma radiation and color reflectance L*^7^, and ice rafted debris (IRD) from Blake-Mizen (2019).

**3. Age models**

**3.1 The age model Blake Mizen et al. (2019) and paleomagnetic anchors**

The age model from Blake-Mizen et al. (2019)^6^ used in Fig. S3 encompasses four paleomagnetic reversals (Gauss-Matuyama, Kaena top, Kaena bottom and Mammoth top) and uses twenty-nine points that link the sedimentary record from site U1307 to the previously dated site U1308^9,10^ from magnetic inclination and relative paleointensity correlations. The age model for site U1308 was established using the benthic oxygen isotope record, which was combined with relative paleointensity data to produce an integrated, high-resolution magneto-isotopic stratigraphy. This age model remains so far the most reliable although differences in the sedimentary rates between glacials/interglacials are not taken into consideration. Fig. S4 depicts the age (ka)/depth (rmcd) relationship using all the 33 anchor points (red line) vs. using only the 4 magnetic anchors (blue line). The offset between the two models may imply a ~40 ka shift at the bottom of the core. This uncertainty is thus large enough to blur correlations with the Earth’s obliquity and precession.

Table S1: Age and uncertainty of paleomagnetic anchor ages.

| Magnetic Reversal | Age (ka) | ±1σ Age (ka) |
| --- | --- | --- |
| Gauss/Matuyama | 2595^10^ | ? |
| Kaena top | 3029^10^ | ? |
| Kaena bottom | 3116^10^ | 15^11^ |
| Mammoth top | 3207^10^ | 15^11^ |

Figure S4: Interpolated ages (ka) vs depth (rmcd) using either the Blake-Mizen et al.'s model (2019)^6^ or the four paleomagnetic anchor ages.

**3.2 Glacials/interglacials intervals of U1307 vs the LR04 stack**


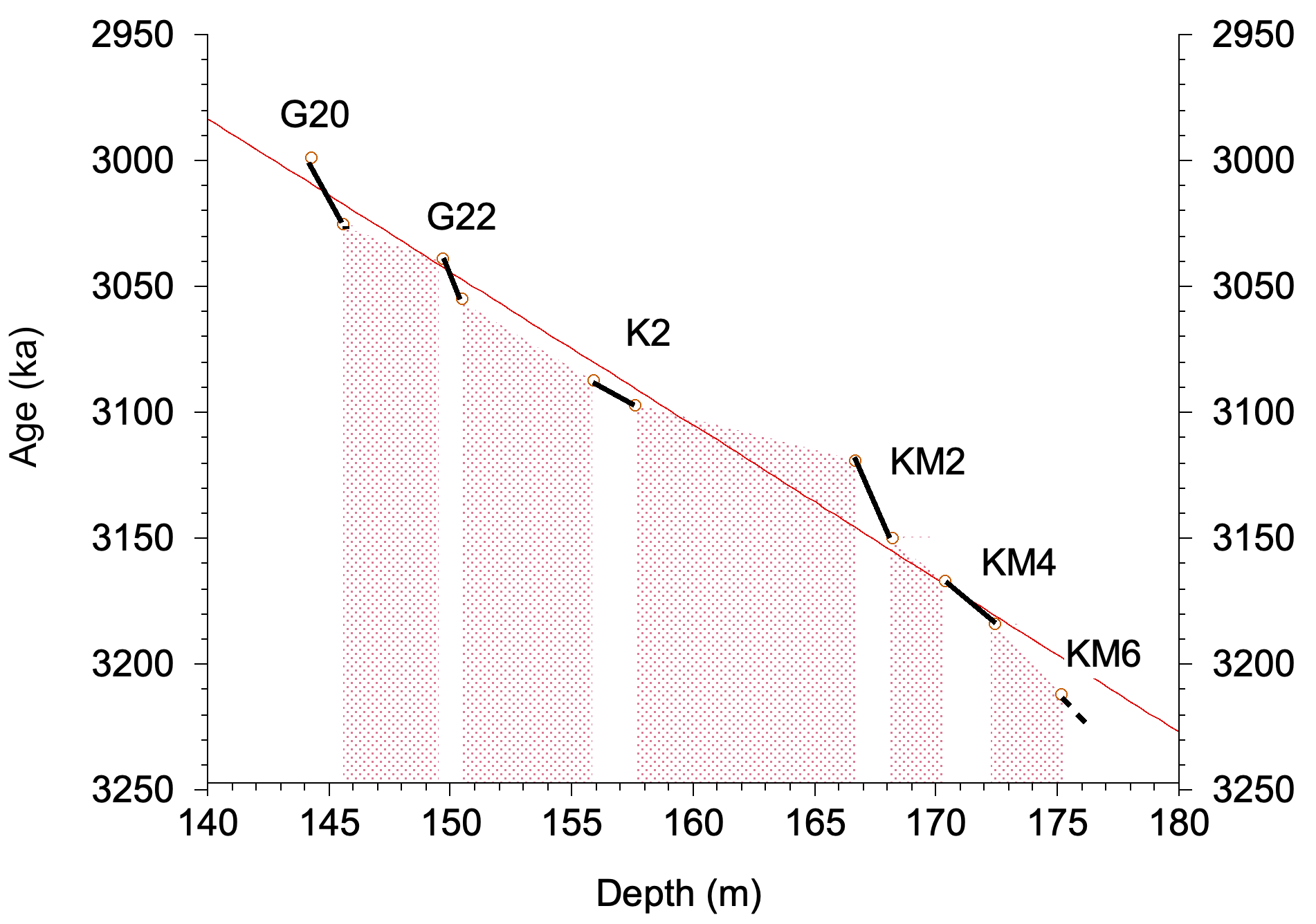
Tentatively, we correlated glacial stages of the Lisiecki and Raymo (2005)^12^ δ^18^O-LR04 stack to glacial intervals of U1307 as identified from Pb isotopic excursions. Note that offsets the stratigraphic correlation with the LR04 stack may generate age model uncertainties of several thousand years during terminations^13^. A simple model demonstrates that these lags can be generated by diachronous temperature changes and do not require slower circulation rates^13^. Hypothesizing an overall slower oceanic circulation during the late Pliocene vs that of the Quaternary, this offset could be even greater as our record illustrates conditions close to major ice masses, thus close to the entry of ^18^O-depleted meltwater into the ocean, whereas the Pliocene benthic δ^18^O-record mostly originates from distal oceanic records.

Figure S5: Tentative correlation of glacial/interglacial boundaries in U1307 (using Pb data) with the LR04 transitions of Lisiecki and Raymo (2005)^12^. Glacials are represented by black segments and numbered following Lisiecki and Raymo (2005)^12^. Higher deposition rates seem recorded during interglacials (colored background) likely in relation with an intensified WBUC.

**3.3 CaCO_3_-free sediment accumulation vs the age models**

The impact of biogenic carbonate fluxes on sedimentation rate has been estimated by comparing the bulk vs carbonate-free sediment accumulations of the investigated sequence. The only significant offset is observed in the upper part of the sequence when the biogenic carbonate is close to 10%. Nevertheless, the cumulative offset between the carbonate-free and bulk sediment accumulation reaches about 20 ka: a value sufficient to blur peak-to-peak correlation of U1307 records with the obliquity cycle, whatever the age model used.


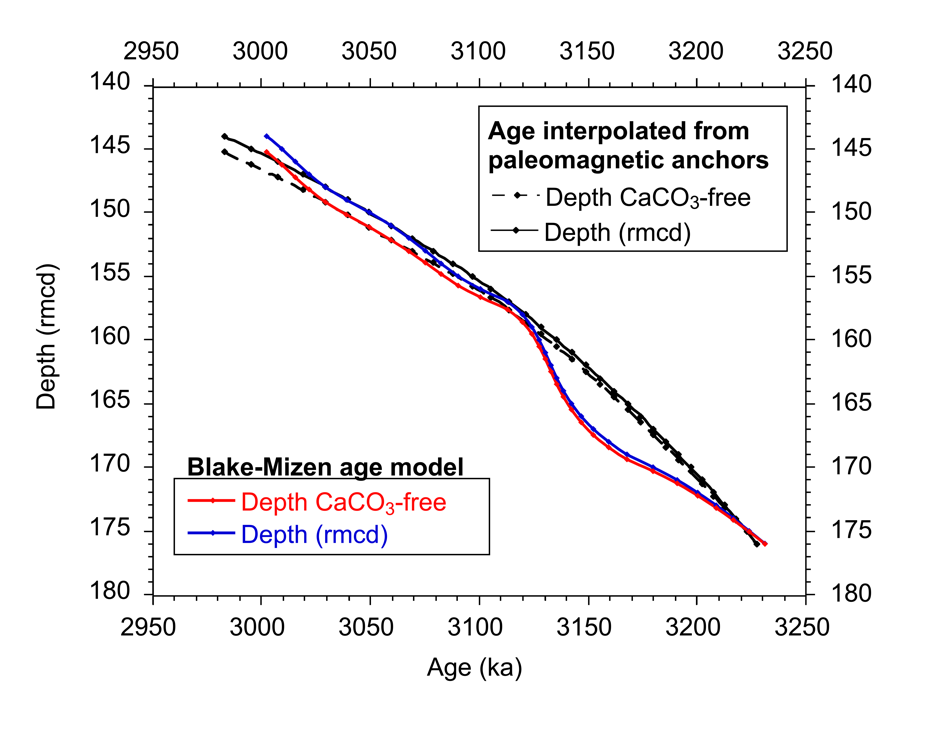


Figure S6: Interpolated ages (ka) vs depth (rmcd) of bulk and carbonate-free sediment.

/

**4. Spectral analysis**

Spectral analyses were performed using the Blake-Mizen et al. (2019) age model. We used the time-series analysis software Acycle 2.8^14^. Nd and Pb isotope data were first interpolated using a sample rate of 3.9 ky, which is the median of the sampling rate. In the periodogram of the ε_Nd(0)_ series, frequencies at which the signal has significant power are 0.021 (47 ky), 0.049 (20 ky) and 0.054 (18 ky). Nonetheless, only the 0.049 frequency exceeds the 99% confidence level. In the ^207^Pb/^206^Pb isotope series, frequencies at which the signal has significant power are 0.021 (47 ka), 0.026 (38 ky), 0.034 (29 ky) and 0.049 (20 ky), but none exceeding the 99% confidence level. The lack of a clear obliquity forcing (~ 41 ka) is possibly a consequence of uncertainties in the age model, as documented above.

Figure S7: Power spectrum of the ε_Nd(0)_ and ^207^Pb/^206^Pb series. Here, bw is the bandwidth.

**Supplementary References**

1. Reyes, A. V. *et al.* South Greenland ice-sheet collapse during Marine Isotope Stage 11. *Nature* **510**, 525–528 (2014).

2. Henriksen, N., Higgins, A. K., Kalsbeek, F. & Pulvertaft, T. C. R. Greenland from Archaean to Quaternary. Descriptive text to the Geological map of Greenland, 1:2 500 000. *Geol. Greenl. Surv. Bull.* **185**, 2–93 (2000).

3. Garde, A. A., Hamilton, M. A., Chadwick, B., Grocott, J. & McCaffrey, K. J. The Ketilidian orogen of South Greenland: geochronology, tectonics, magmatism, and fore-arc accretion during Palaeoproterozoic oblique convergence. *Can. J. Earth Sci.* **39**, 765–793 (2002).

4. Tegner, C., Brooks, C. K., Duncan, R. A., Heister, L. E. & Bernstein, S. 40Ar–39Ar ages of intrusions in East Greenland: Rift-to-drift transition over the Iceland hotspot. *Lithos* **101**, 480–500 (2008).

5. Hillaire-Marcel, C. *et al.* Productivité et flux de carbone dans la mer du Labrador au cours des derniers 40 000 ans. *Can. J. Earth Sci.* **31**, 139–158 (1994).

6. Blake-Mizen, K. *et al.* Southern Greenland glaciation and Western Boundary Undercurrent evolution recorded on Eirik Drift during the late Pliocene intensification of Northern Hemisphere glaciation. *Quat. Sci. Rev.* **209**, 40–51 (2019).

7. Channell, J. E. T., Hodell, D. A. & Curtis, J. H. Relative paleointensity (RPI) and oxygen isotope stratigraphy at IODP Site U1308: North Atlantic RPI stack for 1.2–2.2 Ma (NARPI-2200) and age of the Olduvai Subchron. *Quat. Sci. Rev.* **131**, 1–19 (2016).

8. Ogg, J. G. Geomagnetic Polarity Time Scale. in *Geologic Time Scale 2020* 159–192 (Elsevier, 2020). doi:10.1016/B978-0-12-824360-2.00005-X.

9. Channell, J. E. T., Hodell, D. A., Xuan, C., Mazaud, A. & Stoner, J. S. Age calibrated relative paleointensity for the last 1.5 Myr at IODP Site U1308 (North Atlantic). *Earth Planet. Sci. Lett.* **274**, 59–71 (2008).

10. Channell, J. E. T., Hodell, D. A. & Curtis, J. H. Relative paleointensity (RPI) and oxygen isotope stratigraphy at IODP Site U1308: North Atlantic RPI stack for 1.2–2.2 Ma (NARPI-2200) and age of the Olduvai Subchron. *Quat. Sci. Rev.* **131**, 1–19 (2016).

11. Deino, A. L. *et al.* Chronostratigraphy of the Baringo-Tugen Hills-Barsemoi (HSPDP-BTB13-1A) core – 40Ar/39Ar dating, magnetostratigraphy, tephrostratigraphy, sequence stratigraphy and Bayesian age modeling. *Palaeogeogr. Palaeoclimatol. Palaeoecol.* **570**, 109519 (2021).

12. Lisiecki, L. E. & Raymo, M. E. A Pliocene-Pleistocene stack of 57 globally distributed benthic δ ^18^ O records: PLIOCENE-PLEISTOCENE BENTHIC STACK. *Paleoceanography* **20**, n/a-n/a (2005).

13. Lisiecki, L. E. & Raymo, M. E. Diachronous benthic *δ* ^18^ O responses during late Pleistocene terminations. *Paleoceanography* **24**, 2009PA001732 (2009).

14. Li, M., Hinnov, L. & Kump, L. Acycle: Time-series analysis software for paleoclimate research and education. *Comput. Geosci.* **127**, 12–22 (2019).
