# Supplementary material for: Cold spells over Greenland during the mid-Pliocene Warm Period
Source: Nat Commun. 2025 Feb 22;16:1877. doi: 10.1038/s41467-025-56996-3 (PMC11847007; doi:10.1038/s41467-025-56996-3)
Supplement: Supplementary file 2 — Description of Additonal Supplementary Files [file 41467_2025_56996_MOESM2_ESM.docx]

**Description of Additional Supplementary Files**

File name: Supplementary dataset 1

Description: Grain size measurements

File name: Supplementary dataset 2

Description: Sortable silt

File name: Supplementary dataset 3

Description: Quantitative X-ray diffraction data

File name: Supplementary dataset 4

Description: Radiogenic isotope data
